# Treatment of Child Gratification Disorder 

**DOI:** 10.22037/ijcn.v16i2.35480

**Published:** 2022-03-14

**Authors:** Hamid NEMATI, Farzad AHMADABADI, Mina SHAHISAVANDI, Mohsen FARJOUD KOUHANJANI, Mahtab ROSTAMIHOSSEINKHANI

**Affiliations:** 1 Epilepsy Research Center, Shiraz University of Medical Sciences, Shiraz, Iran.; 2 Pediatric Neurology Research Center, Research Institute for Children’s Health, Shahid Beheshti University of Medical Sciences, Tehran, Iran.; 3 Pediatric Neurology Department, Mofid Children’s Hospital, Faculty of Medicine, Shahid Beheshti University of Medical Sciences, Tehran, Iran; 4 Shiraz Neuroscience Research Center, Shiraz University of Medical Sciences, Shiraz, Iran.; 5 Clinical Neurology Research Center, Shiraz University of Medical Sciences, Shiraz, Iran.

**Keywords:** Self-gratification, Pediatric, Infantile, Masturbation, Gratification Treatment

## Abstract

**Objectives:**

Gratification disorder is a group of self-stimulatory behaviors tending to form a habit. These normal behaviors are common and have various differential diagnoses, including epilepsy. Hence, misdiagnosis may lead to performing unnecessary workups and treatments. In this article, we systematically reviewed available treatment options for gratification disorder.

**Materials & Methods:**

We systematically searched Scopus, MEDLINE, and Embase for related articles published from the beginning to 12^th^ May 2021. We followed the search strategy in all electronic databases using keywords [“Self-gratification” AND “treatment”], [“child” AND “masturbation” AND “treatment”], [“Pediatric” AND “masturbation” AND “treatment”], [“infantile” AND “masturbation” AND “treatment”], and [“Benign” AND “Infantile” AND “Dyskinesia” AND “treatment”].

**Results:**

The primary search yielded 241 studies. Five studies fulfilled the inclusion criteria and were used in the systematic review. None of the studies provided a good level of evidence. These studies indicated that behavioral therapy, escitalopram, and aripiprazole could be considered treatment options.

**Conclusion:**

Although pediatricians are familiar with gratification behaviors, their optimal management is overlooked. In addition to parental education and behavioral therapy, escitalopram and aripiprazole can be used as treatment options for this issue. We need to perform well-designed randomized controlled trials to obtain adequate evidence on the efficacy of these measures.

## Introduction

Infantile and pediatric self-gratification is a benign phenomenon. Gratification disorder, also known as “infantile masturbation” or “benign idiopathic infantile dyskinesia,” comprises a group of self-stimulatory behaviors with a tendency to form a habit (1-4). These movements are often considered “disorders” because they are normal behaviors in early childhood (5, 6). Although masturbation or self-stimulation of the genitalia is typical human behavior, little has been published on gratification disorder (masturbation) in early childhood (1, 7). Misdiagnosis is more probable when there is no apparent genital manipulation, and movements are described as staring, shaking, and moving limbs for minutes (1). Misdiagnosis may result in unnecessary workups and antiepileptic treatments (8-11). 

Masturbation is a non-openly discussed taboo, especially in African societies. Its occurrence in infancy may lead to procedures to control libido and future sexual behaviors, such as female genital cutting (8, 12). Educating and counseling the parents are often the only suggested measures as gratification in infants is usually benign and tends to resolve spontaneously by two years of age (2, 13). Besides, attempts to stop this behavior are not helpful and may even reinforce it (14). Recurrent daily friction in the perineal area may result in irritation. In addition, parents may know this habit as a disease and feel embarrassed if it occurs in public (15, 16).

Sometimes it may result in useless investigations and non-appropriate therapeutic measures. In addition, in some societies, such behaviors are considered a stigma and may be annoying to parents. This study aimed to perform a systematic review to examine proper treatments and family management based on available literature.

## Materials & Methods

**Figure 1:**
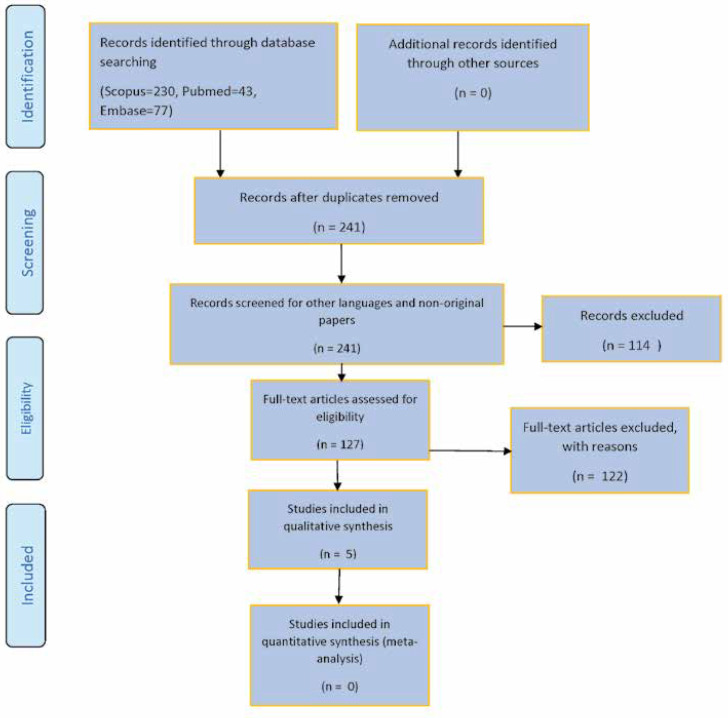
Preferred Reporting Items for Systematic Reviews and Meta-Analyses (PRISMA) Flow Diagram of the study

This study utilized a systematic literature search based on the Preferred Reporting Items for Systematic Reviews and Meta-Analyses (PRISMA) statement (17, 18) (Fig .1). The junior authors (MS, MF, and MR) individually searched electronic databases including Scopus, MEDLINE (from PubMed), and Embase from beginning to 12 May 2021 for related studies. The same search approach was utilized in all electronic databases. The keywords used in the search were [“Self-gratification” AND “treatment”], [“child” AND “masturbation” AND “treatment”], [“Pediatric” AND “masturbation” AND “treatment”], [“infantile” AND “masturbation” AND “treatment”], and [“Benign” AND “Infantile” AND “Dyskinesia” AND “treatment”]. All titles and abstracts were examined to determine the publications' relevance. Exclusion criteria included non-original studies, animal studies, and non-English papers. The authors also checked the reference lists of the included papers or pertinent reviews found in the electronic search. All the three junior authors independently examined the searched sources and participated in all rounds of the review (screening, eligibility, and inclusion). The full texts of all papers meeting the inclusion criteria and any articles in doubt were reviewed. Disagreements about the review were settled after a conversation with the principal author (the first author). All the authors were aware of the journal's titles, authors, or research institutes. All authors assessed the methodological quality of the included studies. The class of evidence was defined following the Levels of Evidence - Wiley Online Library (19) (Appendix 1).

 Standard Protocol Approvals, Registrations, and Patient Consent: 

The Shiraz University of Medical Sciences’ Institutional Review Board approved this systematic review.

**Availability of Data and Material**

Data sharing does not apply to this article.

## Results

The primary search yielded 241 studies. Five studies met the inclusion/exclusion criteria and were used in the systematic review (20-24) (Fig. 1). Table 1 shows a summary of these studies. None of the studies provided a good level of evidence. The summary of related studies indicated that behavioral therapy, escitalopram, and aripiprazole could be possible treatments for this disorder (20-24). Two studies, including one prospective cohort study of 54 patients diagnosed with self-gratification habits, recommended that behavioral therapy could be used for the beneficial management of childhood masturbation (20, 21). Two other studies, as case reports, showed that medications such as escitalopram and aripiprazole are effective for treating child gratification (22, 23). In another case series, two management steps were recommended: 1) reassurance alongside informing the parents about the harmless nature of the activity and 2) managing the anxiety of the children's mothers (24).

## Discussion

This systematic review aimed to examine the management of self-gratification or masturbatory habits. Although these paroxysmal behaviors are prevalent among children, their true prevalence is not addressed in scientific papers (20, 21, 23, 24). A study suggested several diagnostic criteria, including 1) onset after the age of three months and before three years, 2) stereotyped episodes of variable duration, 3) vocalizations with quiet grunting, 4) facial flushing with diaphoresis, 5) pressure on the perineum with characteristic posturing of lower extremities, 6) no alteration of consciousness, 7) cessation with distraction, 8) normal examination, and 9) normal laboratory studies (15). Infantile gratification and masturbation should always be considered a differential diagnosis of epilepsy in infants and children, as well as abdominal pain and movement disorders. A detailed history and video recording are encouraged for a correct diagnosis (8, 20, 25, 26). Possible sexual abuse, genital irritation, familial stress, emotional deprivation, and lack of breastfeeding may positively correlate with childhood masturbation and self-gratification behavior (3, 27- 29). 

 Our systematic review revealed that pediatricians significantly overlook the management of childhood gratification habits. There is no well-designed clinical trial to explore appropriate treatment strategies for childhood gratification habits in the literature. A few existing studies have explored the management of self-gratification habits. This review suggested that despite the benign nature of the entity, due to various religious and cultural taboos, it can cause enormous anxiety and guilty feelings among parents and family members. Therefore, parental education, breastfeeding with tactile stimulus, behavioral therapy, and medications such as escitalopram and aripiprazole, if needed, may help patients with gratification habits (20-24). Even situations that are excluded from medical diagnoses and need no further intervention could be a source of significant distress. “Benign and simply overemphasized form of normal development” could result in severe disarrangement in the mother-child relationship and had to be a target of therapeutic intervention (21). In another study, the authors recommended the instruction of parents to try to interrupt gratification behaviors by distraction and engagement in other activities or plays. Scolding or threatening the infant is not appropriate as efforts to stop the behavior forcefully will only reinforce it and possibly instill a sense of shame or wrong-doing as the infant gets older (13, 30). 

Limited literature is available concerning the long-term follow-up of children with gratification. Only one study with a long-term follow-up (mean 7.1 years) showed that four (21%) children developed features suggestive of Attention Deficit Hyperactivity Disorder (ADHD).

**Table 1:** Summary of the studies

Author /year/country	Method	Result	Limitation	Level of evidence
H. Ayaydın/2018/Turkey [13]	A case repost of a 33-month girl with autism disorder	Escitalopram resolved her masturbatory behavior after three weeks	Case report	4
Biswajit/2020/India [14]	A prospective cohort study of 54 patients diagnosed with self-gratification habits	Sixteen (29.63%) children were referred for behavioral therapy and all of them responded well in next one year.	Small sample size	2b
Franić/2011/Croatia [15]	A case report of a 14-month girl with infantile masturbation	The therapeutic plan was conceived at two levels. The first one was behavioral modification of the child, which was done by distraction of attention during attacks. The second goal was to diminish her mother’s level of anxiety.	Case report	4
Fleisher/1990/USA [16]	Case series of five girls, 7 to 27 months of age, with masturbatory posturing	Management consisted of convincing the parents about the harmless nature of the action, which then lessened the reinforcing effect of their responses.	Case series	4
Kul1/2013/Turkey [17]	A case report of seven-year and two-month old female patients with clinical presentation of childhood masturbation	4 mg aripiprazole resolved her masturbatory behavior after three months	Case report	4

## In Conclusion

Although pediatricians are familiar with gratification behaviors, their optimal management is overlooked. Parental education, behavioral therapy, escitalopram, and aripiprazole can be good candidates for future studies of the management of childhood self-gratification behaviors. We need well-designed, randomized controlled trials to obtain the desired evidence on the efficacy of non-medical or medical management of children with these behaviors. Investigators should calculate the appropriate sample size when designing such clinical trials. 

## Author’s Contribution

Hamid Nemati: Designed and conceptualized the study; analyzed the data; drafted and revised the manuscript. Others: analyzed the data; drafted and revised the manuscript

## Conflict of interest

None
